# Gender disparities among adult recipients of layperson bystander cardiopulmonary resuscitation by location of cardiac arrest in Pan-Asian communities: A registry-based study

**DOI:** 10.1016/j.eclinm.2022.101293

**Published:** 2022-02-12

**Authors:** Nan Liu, Yilin Ning, Marcus Eng Hock Ong, Seyed Ehsan Saffari, Hyun Ho Ryu, Kentaro Kajino, Chih-Hao Lin, Sarah Abdul Karim, G.V. Ramana Rao, Andrew Fu Wah Ho, Shir Lynn Lim, Fahad Javaid Siddiqui, PAROS Clinical Research Network Investigators

**Affiliations:** a Duke-NUS Medical School, National University of Singapore, Singapore, Singapore; b Health Services Research Centre, Singapore Health Services, Singapore, Singapore; c Institute of Data Science, National University of Singapore, Singapore, Singapore; d Department of Emergency Medicine, Singapore General Hospital, Singapore, Singapore; e National Neuroscience Institute, Singapore, Singapore; f Department of Emergency Medicine, Chonnam National University Medical School and Hospital, Gwangju, Republic of Korea; g Department of Emergency and Critical Care Medicine, Kansai Medical University, Moriguchi, Osaka, Japan; h Department of Emergency Medicine, National Cheng Kung University Hospital, College of Medicine, National Cheng Kung University, Tainan, Taiwan; i Department of Emergency Medicine, Hospital Sungai Buloh, Sungai Buloh, Selangor, Malaysia; j GVK Emergency Management and Research Institute (GVK EMRI), Secunderabad, Telangana, India; k Department of Cardiology, National University Heart Centre, Singapore, Singapore; l Department of Medicine, Yong Loo Lin School of Medicine, Singapore, Singapore

**Keywords:** Out-of-hospital cardiac arrest, Gender disparity, Bystander, Emergency medical services, BCPR, Bystander cardiopulmonary resuscitation, CPR, Cardiopulmonary resuscitation, DA-CPR, Dispatcher-assisted CPR, OHCA, Out-of-hospital cardiac arrest, PAROS, Pan-Asian Resuscitation Outcomes Study

## Abstract

**Background:**

Bystander cardiopulmonary resuscitation (BCPR) is a critical component of the 'chain of survival' in reducing mortality among out-of-hospital cardiac arrest (OHCA) victims. Inconsistent findings on gender disparities among adult recipients of layperson BCPR have been reported in the literature. We aimed to fill this knowledge gap by investigating the extent of gender disparities in a cross-national setting within Pan-Asian communities.

**Methods:**

We utilised data collected from the Pan-Asian Resuscitation Outcomes Study (PAROS), an international, multicentre, prospective study conducted between 2009 and 2018. We included all OHCA cases with non-traumatic arrest aetiology transported by emergency medical services and excluded study sites that did not consistently collect information about the location of cardiac arrest. Logistic regression was used to analyse the association between gender and BCPR, stratified by location.

**Findings:**

We analysed a cohort of 56,192 OHCA cases with an overall BCPR rate of 36.2% (20,329/56,192). At public locations, the BCPR rate was 31.2% (631/2022) for female and 36.4% (3235/8892) for male OHCA victims; while at home, the rate was 38.3% (6838/17,842) for females and 35.1% (9625/27,436) for males. Controlling for site differences and several factors in multivariable logistic regression, we found females less likely to receive BCPR than males in public locations (odds ratio [OR]=0.89, 95% confidence interval [CI]: 0.70–0.99), but more likely to receive BCPR at home (OR=1.16, 95% CI: 1.11–1.21).

**Interpretation:**

In Pan-Asian communities, gender differences exist in adult recipients of BCPR and differ between home and public locations. Future studies should account for additional information on bystanders and societal factors to identify targets for interventions.

**Funding:**

The study was supported by grants from the National Medical Research Council (NMRC/CSA/0049/2013) and Laerdal Foundation (20040).

**Research in context**

**Evidence before this study**

We searched PubMed without language restrictions for articles published between January 1, 2010, and January 18, 2021, which reported gender disparity and bystander cardiopulmonary resuscitation (CPR) in out-of-hospital cardiac arrest (OHCA). We used terms ("gender disparit*") AND ("bystander CPR" OR "bystander cardiopulmonary resuscitation") AND ("out-of-hospital cardiac arrest" OR "OHCA"). The majority of studies were based on individual countries, yet the results were inconsistent regarding the gender disparities among adult recipients of layperson bystander cardiopulmonary resuscitation (BCPR) by location of cardiac arrest. In addition, there was little evidence in the Pan-Asian region except for Japan. To our knowledge, the present study is the first to investigate such gender disparities in multiple regions across the Pan-Asian community.

**Added value of this study**

Our findings extended existing knowledge by providing a Pan-Asian perspective involving communities of diverse ethnicities, sociocultural backgrounds and emergency medical services systems-of-care. Our study across 9 Asian communities collected between 2009 and 2018 demonstrated gender disparities in BCPR amongst adult OHCA patients. While women experiencing OHCA in public were less likely to receive BCPR, the converse was observed for OHCA at home. These disparities were more marked in certain sites.

**Implications of all the available evidence**

These findings may provide evidence for future public policy initiatives and the development of CPR training programs tailored to the needs of communities with diverse ethnic and cultural backgrounds.

Alt-text: Unlabelled box

## Introduction

Out-of-hospital cardiac arrest (OHCA) is a serious public health problem across the globe.^1^ Its mortality remains high, despite efforts in refining treatment strategy, raising community awareness, and developing healthcare policy.^2^ Evidence has shown that quick responses and early interventions are vital in caring for OHCA patients.^3^ As a key element in the 'chain of survival', bystander cardiopulmonary resuscitation (BCPR) is one of the earliest possible treatments that an OHCA victim can receive. The landmark OPAL study, a prospective 20-community cohort study, shows that BCPR significantly improved survival and long-term health-related quality of life.^4^

BCPR may be administered by first responders with prior CPR training, healthcare workers or laypersons. However, as opposed to trained personnel, laypersons sometimes may be reluctant to provide CPR due to various reasons, such as emotional stress, inability to recognise cardiac arrest, lack of ability to perform CPR, concerns on causing injury to the patient and fear of accusations of sexual misconduct if the victim is a female.^5^ A public survey in the United States outlined potential barriers to performing BCPR on women in the general public, including concerns about inappropriate touching and allegations of sexual assault.^6^

There has been evidence of gender disparities among recipients of layperson BCPR, but results have been inconsistent.^7^ Several studies showed that adult women were less likely to receive BCPR than men in public places,^8^^,^^9^ while others reported no significant differences.^10^ For OHCA events at home, a study reported no gender difference in receiving layperson BCPR^9^ but another reported a higher chance of layperson BCPR for women than men.^10^ An earlier systematic review suggested that women were more likely to receive bystander CPR among witnessed OHCA.^11^ Given the discordance of evidence available, it is worthwhile to investigate further gender disparities among adult BCPR recipients. Such investigation is necessary to ensure that strategies and policies designed to increase BCPR benefit both genders equally.

The majority of research on gender disparities in BCPR recipients has been conducted based on individual countries such as the United States,^9^ Japan,^10^ and the Netherlands.^8^ There is a lack of evidence to determine the extent of gender disparities in a cross-national setting, particularly in Pan-Asian communities, which present a wide range of social and cultural characteristics, population traits, and emergency medical systems (EMS).^12^ This study aimed to investigate gender disparities in receiving BCPR among adult OHCA victims in the Pan-Asian region. The research hypothesis is that adult women would be less likely than men to receive BCPR in public and have comparable chances of receiving BCPR at home.

## Methods

### Study design and setting

We performed a secondary analysis of the Pan-Asian Resuscitation Outcomes Study (PAROS), an international, multicentre, prospective trial conducted between 2009 and 2018. PAROS, founded in 2010, is an Asia-based multi-national clinical research network for OHCA. The PAROS registry includes sites in Singapore, Japan, Korea, Thailand, Taiwan, Malaysia, China, Philippines, Vietnam, Pakistan, India, Lebanon, and the United Arab Emirates (UAE). A common taxonomy and case report form were developed to standardise data collection and recording. The details of the network have previously been published.^12^ In addition to patient-related variables (such as age and gender), incident-related variables (such as bystander CPR, witness status, location type) and EMS-related variables (such as response time) are also collected. The PAROS registry includes both urban and rural communities with a variety of EMS systems.^12^ The study was granted approval from SingHealth Centralised Institutional Review Board and Domain Specific Review Board with a waiver of patient informed consent.

### Patient selection

Adult OHCA patients (≥18 years) in the PAROS registry with non-traumatic aetiology transported by EMS were included in the study. OHCA is defined by the absence of pulse, unresponsiveness, and apnoea. CPR is considered present if chest compressions are done. Sites were excluded if they did not consistently collect information regarding the location of cardiac arrest. In the remainder of the paper, BCPR is referred to as a layperson BCPR, which includes dispatcher-assisted CPR (DA-CPR) performed by layperson bystanders in all sites. Non-layperson BCPR cases—defined as bystander CPR performed by healthcare providers in healthcare facilities, nursing homes or witnessed by EMS/private ambulance crews, were excluded. In addition, patients who had no attempted resuscitation, had been pronounced dead on the scene, or had incomplete records were excluded. We then excluded sites with less than 100 eligible cases, as they are not representative of the corresponding areas and would cause data sparsity issues in subsequent multivariable analyses. A public place is designated as being a public or commercial building, a street or highway, an industrial area, a transportation centre, or a place of recreation in connection with a cardiac arrest.

### Statistical analysis

Categorical variables were presented as frequency and percentage, while continuous variables were reported as median, 1st and 3rd quartile statistics. Patient characteristics were reported for the entire cohort as well as by location (Public and Home) and by layperson BCPR status.

Multivariable logistic regression analysis was performed to investigate the association of gender and layperson BCPR. Adjusted odds ratio (OR) and 95% confidence interval (CI) were calculated for the entire population and by location (public place vs. home), controlling for site, age, witnessed arrest, time from call received to EMS arrival at scene (response time) and time of the day of arrest that have been observed to associate with BCPR in previous studies.^7^^,^^9^^,^^10^ In a secondary analysis, we examined whether layperson BCPR or gender was associated with the probability of survival to discharge (or to 30 days if not yet discharged), accounting for site, age, initial rhythm and response time. Due to the low survival rate, Firth's penalised likelihood method was used in the multivariable logistic regression to control for bias.^13^ Age was analysed as a continuous variable in all adjusted analyses to avoid residual confounding that may arise from categorising continuous confounders. The significance level was set at *p* < 0.05. The data were analysed using R software, version 4.0.5 (R Foundation for Statistical Computing).

### Role of the funding source

The funders were not involved in the study design, collection, analysis, and interpretation of data, nor did they have a role in the writing of the paper and decision to submit the paper for publication. All authors had access to the data in the study and had final responsibility for the decision to submit for publication.

## Results

### Characteristics of OHCA events

**Figure 1:**
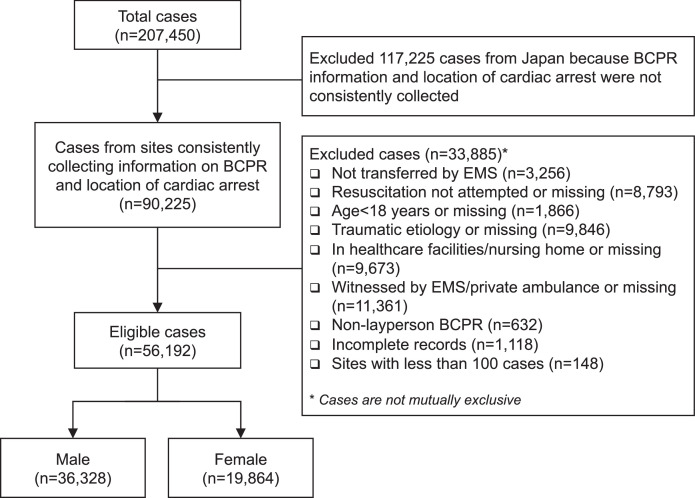
Flow chart for patient selection.

Out of 207,450 OHCAs in the PAROS registry, we included 56,192 (27.1%) for analysis (Figure 1). Considering a critical variable, the location of arrest, was missing from all cities of Japan except Osaka, we excluded these sites from the analysis. The OHCAs included were from Japan (Osaka city), Korea (Seoul, Daegu, and Gwangju), Malaysia (Kuching, Klang Valley, Kota Bahru, Miri, and Penang), Singapore (Singapore), Taiwan (Tainan, Taipei, and Taoyuan), Thailand (Bangkok, and Songkla), UAE (Dubai), China (Zhejiang province), and India (Telangana).

Patient characteristics are detailed in Table 1, and a comparison with the 141,160 cases excluded from the study can be found in Supplementary Table 1. Overall BCPR rate was 36.2% (20,329/56,192) among the total cohort, and the rate differed among sites: 38.3% (5221/13,636) for Japan, 49.1% (6243/12,711) for Korea, 30.2% (430/1424) for Malaysia, 43.4% (4167/9606) for Singapore, 27.2% (4012/14,725) for Taiwan, 18.6% (195/1046) for Thailand, 4.7% (16/342) for UAE and 6.5% (45/696) for China. Among the 56,192 OHCAs analysed, none of the 2006 cases from India had BCPR.

**Table 1:** Patient demographic characteristics.

	All (*n* = 56,192)	Public (*n* = 10,914, 19.4%)			Home (*n* = 45,278, 80.6%)		
		Layperson BCPR (*n* = 3866, 35.4%)	No BCPR (*n* = 7048, 64.6%)	p-value*	Layperson BCPR (*n* = 16,463, 36.4%)	No BCPR (*n* = 28,815, 63.6%)	p-value*
**Gender, No. (%)**				<0.001			<0.001
Female	19,864 (35.4)	631 (16.3)	1391 (19.7)		6838 (41.5)	11,004 (38.2)	
Male	36,328 (64.6)	3235 (83.7)	5657 (80.3)		9625 (58.5)	17,811 (61.8)	
**Age, median (Q1-Q3)**	70 (57, 81)	60 (50, 70)	60 (49, 70)	0.180	72 (59, 82)	73 (60, 83)	<0.001
**Age, No. (%)**				0.165			<0.001
18–29	1255 (2.2)	142 (3.7)	303 (4.3)		332 (2.0)	478 (1.7)	
30–39	2389 (4.3)	263 (6.8)	496 (7.0)		664 (4.0)	966 (3.4)	
40–49	4624 (8.2)	546 (14.1)	1000 (14.2)		1080 (6.6)	1998 (6.9)	
50–59	8213 (14.6)	963 (24.9)	1639 (23.3)		2114 (12.8)	3497 (12.1)	
60–69	10,450 (18.6)	893 (23.1)	1663 (23.6)		2964 (18.0)	4930 (17.1)	
70–79	13,097 (23.3)	667 (17.3)	1288 (18.3)		3996 (24.3)	7146 (24.8)	
80+	16,164 (28.8)	392 (10.1)	659 (9.4)		5313 (32.3)	9800 (34.0)	
**Arrest location, No. (%)**#				<0.001			–
Home residence	45,278 (80.6)	–	–		16,463 (100)	28,815 (100)	
Public/Commercial building	3984 (7.1)	1443 (37.3)	2541 (36.1)		–	–	
Street/highway	2532 (4.5)	514 (13.3)	2018 (28.6)		–	–	
Industrial area	813 (1.4)	335 (8.7)	478 (6.8)		–	–	
Transport center	256 (0.5)	82 (2.1)	174 (2.5)		–	–	
Place of recreation	687 (1.2)	398 (10.3)	289 (4.1)		–	–	
Other	2642 (4.7)	1094 (28.3)	1548 (22.0)		–	–	
**Time of day, No. (%)**				<0.001			<0.001
11:00 pm - 5:59 am	10,268 (18.3)	337 (8.7)	1014 (14.4)		3247 (19.7)	5670 (19.7)	
6:00 am - 6:59 pm	35,881 (63.9)	2922 (75.6)	4929 (69.9)		9981 (60.6)	18,049 (62.6)	
7:00 pm – 10:59 pm	10,043 (17.9)	607 (15.7)	1105 (15.7)		3235 (19.7)	5096 (17.7)	
**Witness, No. (%)**	24,714 (44.0)	2619 (67.7)	2926 (41.5)	<0.001	7814 (47.5)	11,355 (39.4)	<0.001
**Response time (minutes), median (Q1-Q3)**	7.0 (5.0, 9.0)	7.0 (5.0, 9.3)	7.0 (5.0, 10.6)	<0.001	6.5 (5.0, 9.0)	7.0 (5.0, 9.0)	<0.001
**Initial rhythm, No. (%)**							<0.001
Shockable	6839 (12.2)	1379 (35.7)	1459 (20.7)		1873 (11.4)	2128 (7.4)	
Unshockable	43,835 (78)	2247 (58.1)	3966 (56.3)		13,845 (84.1)	23,777 (82.5)	
Cannot determine	5518 (9.8)	240 (6.2)	1623 (23)		745 (4.5)	2910 (10.1)	
**ROSC at ED, No. (%)**	15,346 (27.3)	1682 (43.5)	2011 (28.5)	<0.001	4566 (27.7)	7087 (24.6)	<0.001
**Survival at discharge or in hospital at day 30, No. (%)**	3682 (6.6)	795 (20.6)	719 (10.2)	<0.001	942 (5.7)	1226 (4.3)	<0.001
**Site, No. (%)**				<0.001			<0.001
Japan	13,636 (24.3)	992 (25.7)	1621 (23)		4229 (25.7)	6794 (23.6)	
Korea	12,711 (22.6)	1311 (33.9)	1346 (19.1)		4932 (30)	5122 (17.8)	
Malaysia	1424 (2.5)	80 (2.1)	193 (2.7)		350 (2.1)	801 (2.8)	
Singapore	9606 (17.1)	765 (19.8)	904 (12.8)		3402 (20.7)	4535 (15.7)	
Taiwan	14,725 (26.2)	673 (17.4)	1354 (19.2)		3339 (20.3)	9359 (32.5)	
Thailand	1046 (1.9)	19 (0.5)	127 (1.8)		176 (1.1)	724 (2.5)	
UAE	342 (0.6)	11 (0.3)	133 (1.9)		5 (0)	193 (0.7)	
China	696 (1.2)	15 (0.4)	126 (1.8)		30 (0.2)	525 (1.8)	
India	2006 (3.6)	0 (0)	1244 (17.7)		0 (0)	762 (2.6)	

Q1-Q3, first- third-quartile; EMS, emergency medical services.

Cities and regions enrolled in PAROS study: Osaka city of Japan; Seoul, Daegu, and Gwangju of Korea; Kuching, Klang Valley, Kota Bahru, Miri, and Penang of Malaysia; Singapore of Singapore; Tainan, Taipei, and Taoyuan of Taiwan; Bangkok, and Songkla from Thailand; Dubai of UAE; Zhejiang province of China; Telangana of India.

⁎ p-values for comparison between BCPR and no BCPR, from the Chi-square test for categorical variables and Mann-Whitney test for continuous variables.

# Some communities do not have complete location data.

BCPR rate was similar among OHCAs occurred at home (36.4%; 16,463/45,278) and at public locations (35.4%; 3866/10,914). More specifically, in public locations, 31.2% (631/2022) of female and 36.4% (3235/8892) of male OHCA victims received BCPR. At home, 38.3% (6838/17,842) of female and 35.1% (9625/27,436) of male OHCA victims received BCPR.

### Unadjusted analysis of BCPR

**Figure 2:**
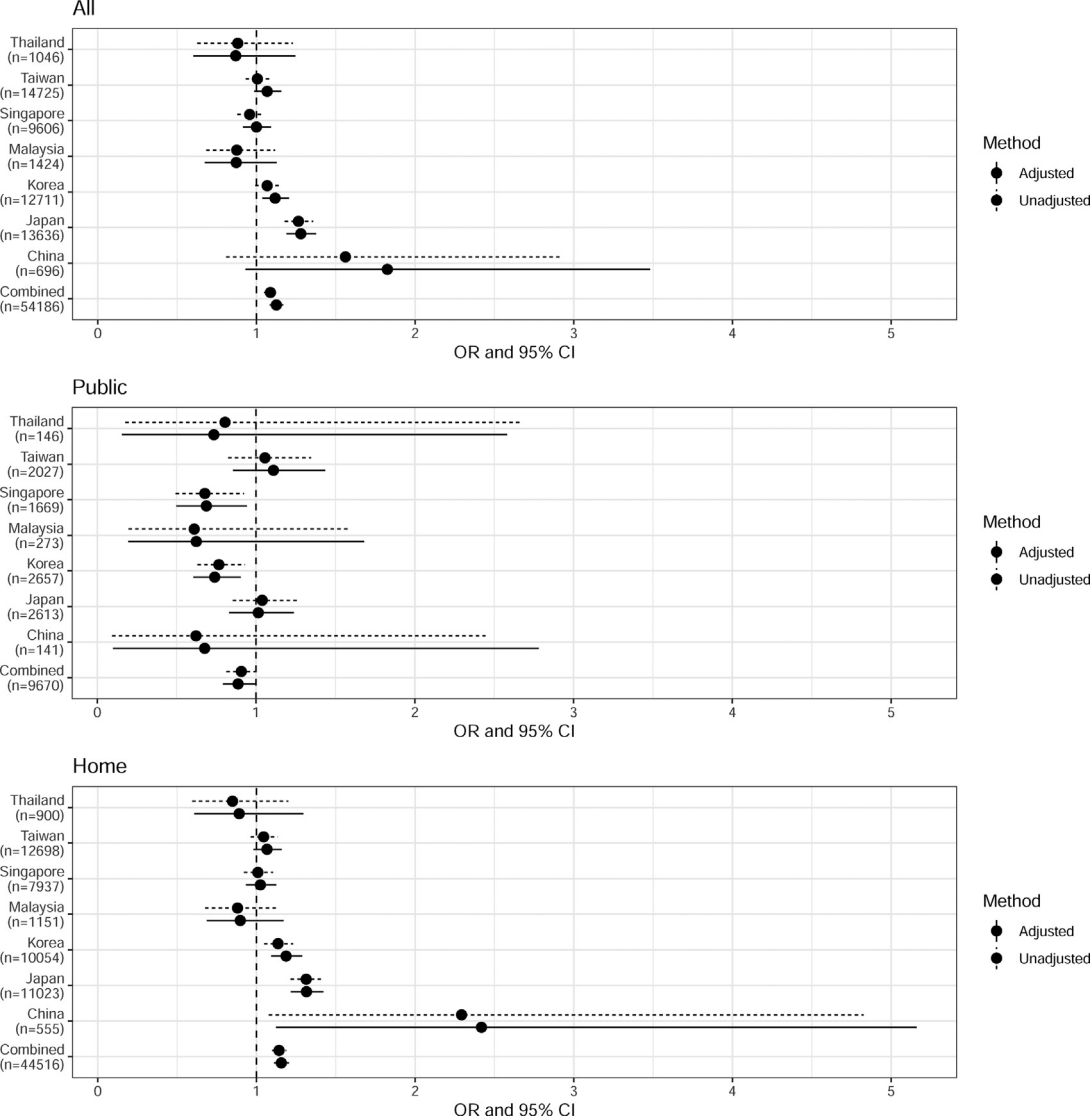
Estimated odds ratio (OR) and the 95% confidence interval (CI) of receiving layperson BCPR for female OHCA victims from unadjusted and adjusted logistic regression analyses stratified by sites (reference: male OHCA victims). Age, time of day and witness status were included in the adjusted logistic regression models. When analysing cases in all locations the model further adjusted for the location of OHCA (public or home). None of the OCHA victims from India had layperson BCPR; hence it was not included in the figure. UAE was not included in the figure due to wide 95% CI: in unadjusted analyses the estimated OR was 1.61 (95% CI: 0.44–4.81) in all locations, 9.63 (95% CI: 1.16–65.83) in public locations and 1.76 (95% CI: 0.23–10.91) at home; in adjusted analyses the estimated OR was 4.82 (95% CI: 1.02–23.21) in all locations, 12.14 (95% CI: 0.92–296.68) in public locations and 2.77 (95% CI: 0.32–19.44) at home. Cities and regions enrolled in PAROS study: Osaka city of Japan; Seoul, Daegu, and Gwangju of Korea; Kuching, Klang Valley, Kota Bahru, Miri, and Penang of Malaysia; Singapore of Singapore; Tainan, Taipei, and Taoyuan of Taiwan; Bangkok, and Songkla from Thailand; Dubai of UAE; Zhejiang province of China; Telangana of India.

The unadjusted analysis found female OHCA victims more likely to receive BCPR in all locations (OR=1.09, 95% CI: 1.05 – 1.13) and at home (OR=1.14, 95% CI: 1.10–1.19), and did not find a gender difference in public locations (OR=0.91, 95% CI: 0.81 – 1.01). These ORs were visualised in Figure 2 in rows labelled “Combined”. However, gender disparity in BCPR rate differed when the unadjusted analysis was stratified by sites (see Figure 2). None of the OHCA victims from India received layperson BCPR. The higher overall BCPR rate for female OHCA victims was only observed from Korea and Japan, while in other sites there was no significant gender difference. In public locations, data from Singapore and Korea suggested a lower BCPR rate for female OHCA victims, and no gender disparity was observed in other sites. Gender disparity at home was only observed in Korea, Japan and China, where females had a higher BCPR rate than males. Due to the small sample size from UAE (342 in all locations, with 144 in public locations and 198 at home), the ORs had much wider 95% CIs than for other sites and were generally not reliable for inference (see footnote of Figure 2). When adjusted for age, time of day and witness status (and location when studying cases in all locations), the site-specific ORs were similar to the unadjusted ones, except for UAE that had unstable estimates due to data sparsity (see Figure 2). We accounted for this difference in sites in the adjusted analyses, where all 2006 OHCA cases from India that had no layperson BCPR were excluded.

### Multivariable logistic regression analysis of BCPR

In the adjusted analysis of 54,186 cases, the likelihood of receiving layperson BCPR was higher at home than in public locations. After adjusting for site difference and other factors females were less likely to receive layperson BCPR than male among cases occurred in public places (OR=0.89, 95% CI: 0.70–0.99), but more likely to receive layperson BCPR at home (OR=1.16, 95% CI: 1.11 – 1.21; see Table 2 and rows in Figure 2 labelled “Combined”).

**Table 2:** Multivariable logistic regression analysis of probability of receiving layperson BCPR.

	All (*n* = 54,186)			Public (*n* = 9670)			Home (*n* = 44,516)		
	OR (95% CI)	Global p-value	Individual p-value	OR (95% CI)	Global p-value	Individual p-value	OR (95% CI)	Global p-value	Individual p-value
**Female**	1.13 (1.08, 1.17)		<0.001	0.89 (0.79, 0.99)		0.039	1.16 (1.11, 1.21)		<0.001
**Age (per 10 year)**	0.95 (0.94, 0.96)		<0.001	0.96 (0.93, 0.98)		0.002	0.95 (0.94, 0.96)		<0.001
**Time of day (baseline: 11:00 pm - 5:59 am)**		<0.001			<0.001			<0.001	
6:00 am - 6:59 pm	1.05 (1.00, 1.10)		0.064	1.46 (1.26, 1.69)		<0.001	0.99 (0.94, 1.04)		0.634
7:00 pm – 10:59 pm	1.15 (1.08, 1.22)		<0.001	1.43 (1.20, 1.69)		<0.001	1.11 (1.04, 1.18)		0.001
**Witness**	1.46 (1.41, 1.52)		<0.001	2.15 (1.97, 2.35)		<0.001	1.33 (1.28, 1.39)		<0.001
**Public location**	1.01 (0.96, 1.06)		0.652	–		–	–		–
**Site (baseline: Singapore)**		<0.001			<0.001			<0.001	
Japan	0.88 (0.84, 0.93)		<0.001	0.86 (0.75, 0.98)		0.021	0.89 (0.84, 0.94)		<0.001
Korea	1.29 (1.22, 1.36)		<0.001	1.29 (1.14, 1.47)		<0.001	1.30 (1.23, 1.38)		<0.001
Malaysia	0.50 (0.45, 0.57)		<0.001	0.45 (0.34, 0.59)		<0.001	0.52 (0.46, 0.60)		<0.001
Taiwan	0.53 (0.50, 0.56)		<0.001	0.63 (0.55, 0.72)		<0.001	0.51 (0.48, 0.54)		<0.001
Thailand	0.28 (0.24, 0.33)		<0.001	0.17 (0.10, 0.27)		<0.001	0.31 (0.26, 0.37)		<0.001
UAE	0.06 (0.04, 0.10)		<0.001	0.10 (0.05, 0.17)		<0.001	0.03 (0.01, 0.07)		<0.001
China	0.08 (0.06, 0.11)		<0.001	0.12 (0.07, 0.20)		<0.001	0.07 (0.05, 0.10)		<0.001

CI, confidence interval; OR, odds ratio.

Global p-values were assessed using likelihood ratio tests.

Cities and regions enrolled in PAROS study: Osaka city of Japan; Seoul, Daegu, and Gwangju of Korea; Kuching, Klang Valley, Kota Bahru, Miri, and Penang of Malaysia; Singapore of Singapore; Tainan, Taipei, and Taoyuan of Taiwan; Bangkok, and Songkla from Thailand; Dubai of UAE; Zhejiang province of China; Telangana of India. This analysis excludes all 2006 OHCA cases from India that had no layperson BCPR cases.

Increased age was found to be associated with decreased probability of receiving layperson BCPR in all locations (overall OR=0.95, 95% CI: 0.94–0.96 per 10-year increase in age), and the effect was similar in public locations and at home. We tested for interaction effect between gender and age in sensitivity analyses but did not find the effect significant in overall or location-stratified analyses.

### Survival after OHCA events

We studied the survival probability of these patients at discharge (or day 30 of inpatient stay if not yet discharged) for OHCA cases in all arrest locations (i.e., in public locations and at home), using data from all 56,192 OHCA victims (including those from India). The overall survival rate was 6.5% (3682/56,192). In a multivariable Firth logistic regression including gender, receiving BCPR, site, and response time, we found receiving BCPR was associated with a higher probability of 30-day survival (OR=1.52, 95% CI: 1.41–1.62) and females were associated with a lower likelihood of survival (OR=0.63, 95% CI: 0.58–0.68). Adjustment for age (years) reduced the estimated effect of gender (OR=0.72, 95% CI: 0.67–0.78) but had little impact on the estimated effect of receiving BCPR (OR=1.47, 95% CI: 1.37–1.58). However, gender difference was no longer significant with further adjustment for initial rhythm (OR=0.95, 95% CI: 0.87–1.03), and the estimated effect of receiving BCPR reduced (OR=1.32, 95% CI: 1.22–1.42).

## Discussion

Our study across 9 Asian communities demonstrated gender disparities in BCPR amongst adult OHCA patients. While women experiencing OHCA in public were less likely to receive BCPR (BCPR rate was 31.2%, compared to 36.4% in men), the converse was observed for OHCA at home (BCPR rate was 38.3% in women and 35.1% in men). These disparities were more marked in certain sites. Findings from some parts of the world are consistent with our results, where women were found to be at a disadvantage regarding receiving BCPR in public locations.^8^^,^^9^^,^^14^ Interestingly, some Asian studies (ours and a study using All-Japan Ustein Registry^10^) indicate that women are more likely to receive BCPR in private locations. Our findings extended existing knowledge by providing a Pan-Asian perspective involving communities of diverse ethnicities, sociocultural backgrounds and EMS systems-of-care.

Equity in care, particularly in terms of gender, has been of considerable concern in OHCA for quite some time.^15^ Data from the US^9^ reported 39% of women and 45% of men received BCPR, while evidence from Japan^10^ shows 54.2% of women and 57% of men received BCPR in public locations. According to both studies, the difference in the prevalence of the receipt of BCPR between men and women OHCA victims in public places was 6% and 2.8%, respectively. In our study, this difference was 5.2%. The variations in BCPR rates across multiple communities could be attributed to several factors, including how the BCPR, the bystander, and the public location were defined. Subtle differences in each of these will have an impact on the prevalence we observed. For example, Blewer et al.^9^ did not mention whether BCPR definition included DA-CPR or mouth-to-mouth breathing. Matsuyama et al.^10^ mentioned information collection on DA-CPR but did not specify whether this was part of the definition. Our study considered a BCPR to be present if chest compressions were done, which included DA-CPR.

The difference in the receipt of BCPR between men and women in public places may be influenced, at least in part, by the cultural beliefs, practices, and laws prevalent in a particular community. Perman et al. conducted an in-depth survey of perceptions among responders regarding why women are less likely to receive BCPR.^6^ Three main themes were identified in this study: (a) Sexualisation of women's bodies; (b) women are frail and prone to injury; and (c) misperceptions about women in acute medical distress. Bouland et al. listed 'Fear of being sued' as one of the barriers to conducting CPR.^16^ Such gender differences extend to AED application, which, for example, has been observed in Japan despite children being familiarised with CPR in schools.^17^^,^^18^

While several studies have investigated gender differences in the receipt of BCPR interventions, they represented EMS systems governed by a single policy and a relatively homogenous public attitude toward cardiac emergencies.^9^^,^^10^^,^^14^ Additionally, there are no systematically conducted studies in most parts of the Pan-Asian region. Our study filled a knowledge gap that existed in the literature. It consisted of a heterogeneous set of EMS systems, some of which covered the entire country, others a single city. There was considerable variation in level of development between each of the EMS systems and the emphasis placed on community intervention and its awareness, resulting in a wide range of BCPR rates across Asia.^19^

Several factors may be associated with gender differences in receiving BCPR. Okubo et al.^17^ reported higher odds of women receiving BCPR if the bystander is a family member. The lower prevalence of women experiencing BCPR in public might be influenced by the relationship between the victim and the bystander. In addition, we were unable to find any studies examining BCPR when both bystanders and victims were female. Perman et al.^6^ found that most of their survey responders assumed the situation of 'a man giving CPR to a woman'. In addition to the bystander factor, a recent study by Ko et al.^20^ demonstrated the value of DA-CPR in reducing gender disparities among BCPR recipients. With a large number of OHCAs occurring worldwide, a reduction in gender disparity (i.e., increased BCPR for women victims) may result in a higher number of women receiving necessary life-saving treatment.^21^

Beyond disparities in BCPR, gender disparity in OHCA has been an active area of clinical research.^22^, ^23^, ^24^, ^25^ Differences have been observed in all elements of an OHCA case, ranging from patient- and event-related characteristics, care provision, to patient outcomes.^7^^,^^11^ Literature has shown that male patients were younger and had a higher probability of witnessed arrest and shockable initial rhythm.^7^^,^^8^ They were also more likely to get advanced life support or targeted interventions (e.g., epinephrine, advanced airway management).^26^^,^^27^ These sex-related differences are associated with a disparity in patient outcomes. On the one hand, women were observed to have a better chance of surviving to hospital admission.^7^ However, females generally achieved equal or poorer overall survival,^8^^,^^28^^,^^29^ except in certain sub-cohorts, for instance, in women of reproductive age or with shockable initial rhythm.^23^^,^^30^ Our study found no significant gender difference in survival after adjusting for other confounders, whereas some American^9^ and European^8^ studies suggested that males have a greater chance of survival than females. Considering these inconsistent findings, further studies are required to investigate gender disparities by better accounting for the cultural, geographical, and ethnic factors.

Our study has a few limitations. First, we could not include all of the PAROS sites in this analysis because the required level of high-resolution information was not available from all of them. More than 72% (151,258/207,450) of PAROS data were not included in this analysis, where 10,098 of these cases were not eligible because they occurred in healthcare facilities or nursing homes or received CPR from healthcare professionals, and the remaining 141,160 cases were excluded due to missing information on key variables of interest, e.g., location information that was missing from all sites in Japan except Osaka city. Although differences were detected between the 56,192 cases included and the 141,160 cases excluded due to missing information for all variables of interest except for witness status (Supplementary Table 1), these factors have been controlled for in the adjusted analyses to reduce bias when assessing gender differences. Second, the cases from some geographic areas may not reflect the overall situation in the whole country, thus affecting the generalizability of our results. In particular, none of the Indian patients in the PAROS registry received BCPR, representing a pronounced bias in the data collection process. Despite this, the inclusion of their data revealed significant heterogeneity among sites and provided evidence indicating a compelling reason to study this aspect within sites. Lastly, the information collected in the PAROS database concerning layperson witnesses does not differentiate between passers-by and employees/professionals (e.g., police, security guards, station personnel). Moreover, the gender of the bystander was not recorded in the registry, which could have provided valuable insight. More qualitative research may be helpful to ascertain additional dimensions of the topic.

Gender differences among adult recipients of BCPR in public exist in Pan-Asian communities. Regardless of age, time of day, or witnessed status, women experienced lower BCPR rates in public than when the arrest occurred at home. Additional factors associated with the outcome, such as gender and relationship of bystander with the OHCA victim, as well as societal factors (e.g., laws and attitudes), need to be assessed to identify targets for interventions.

## Data sharing statement

All data are stored in a secure server environment hosted by Singapore Clinical Research Institute and can be accessed by researchers in the Pan-Asian Resuscitation Outcomes Study (PAROS) Clinical Research Network. For further information, please contact Dr Nan Liu (liu.nan@duke-nus.edu.sg).

Participating site investigators: H Tanaka (Graduate School of EMS System, Kokushikan University, Tokyo, Japan); Tagami T (Nippon Medical School Tama Nagayama Hospital, Tokyo, Japan); T Nishiuchi (Department of Acute Medicine, Kindai University Faculty of Medicine, Japan); SD Shin (Department of Emergency Medicine, Seoul National University College of Medicine, Seoul, Republic of Korea); HW Ryoo (Department of Emergency Medicine, Kyungpook National University Hospital, Daegu, Korea); MHM Ma (Department of Emergency Medicine, National Taiwan University Hospital Yunlin Branch, Douliou, Taiwan); PCI Ko (Department of Emergency Medicine, National Taiwan University Hospital, College of Medicine, National Taiwan University, Taipei, Taiwan); CW Kuo (Department of Emergency Medicine, Chang-Gung Memorial Hospital, Linkou, Taoyuan, Taiwan); P Khruenkarnchana (International Medical Services, Bangkok Hospital, Bangkok, Thailand); J Supasaowapak (Rajavithi Hospital, Bangkok, Thailand); KD Wong (Emergency Department, Hospital Pulau Pinang, Penang, Malaysia); NE Doctor (Sengkang General Hospital, Singapore); S Arulanandam (Emergency Medical Services Department, Singapore Civil defence Force, Singapore); HN Gan (Changi General Hospital, Singapore); BSH Leong (National University Hospital, Singapore); SO Cheah (Urgent Care Clinic International, Singapore); WM Ng (Ng Teng Fong General Hospital, Singapore); DR. Mao (Khoo Teck Puat Hospital, Singapore); YY Ng (Tan Tock Seng Hospital, Singapore); LP Tham (KK Women's & Children's Hospital, Singapore); R Rao (GVK Emergency Management and Research Institute, Telangana, India); M Vimal (GVK Emergency Management and Research Institute, Telangana, India); FJ Gaerlan (Southern Philippines Medical Center, Davao, Philippines); W Cai (Zhejiang Provincial People's Hospital, Zhejiang, China); SA Zhou (Zhejiang Provincial People's Hospital, Zhejiang, China); M Khursheed (Emergency Department, National Institute of Cardiovascular Diseases, Karachi, Pakistan), DA Nguyen (Bach Mai Hospital, Hanoi, Vietnam); S AlQahtani (National Ambulance, Abu Dhabi, United Arab Emirates); O Al Sakaf (Department of Medical and Technical Affairs, Dubai Corporation for Ambulance Services, Dubai, United Arab Emirates); AL Blewer (Duke University School of Medicine, North Carolina, United States of America).

NL conceived and designed the study. NL, YN, SES, and FJS accessed and verified the underlying study data. NL, YN, SES, and FJS analysed the data. All authors interpreted the data. NL, YN, SES, and FJS drafted the manuscript. All authors critically revised the manuscript for intellectual content. NL, MEHO, and FJS supervised the study. All authors had access to all the data in the study and had final responsibility for the decision to submit for publication.

The study was supported by grants from the 10.13039/501100001349National Medical Research Council (NMRC/CSA/0049/2013) and 10.13039/501100004102Laerdal Foundation (20040).

## Declaration of interests

MEH Ong reports funding from the ZOLL Medical Corporation for a study involving mechanical cardiopulmonary resuscitation devices; grants from the Laerdal Foundation, Laerdal Medical, and Ramsey Social Justice Foundation for funding of the Pan-Asian Resuscitation Outcomes Study; an advisory relationship with Global Healthcare SG, a commercial entity that manufactures cooling devices; and funding from Laerdal Medical on an observation program to their Community CPR raining centre Research Program in Norway. MEH Ong has a licensing agreement and a patent filed (Application no: 13/047,348) with ZOLL Medical Corporation for a study titled "Method of predicting acute cardiopulmonary events and survivability of a patient. All other authors have no conflict of interests to declare.

## Appendix Supplementary materials

Image, application 1
