# Supplementary material for: Gender disparities among adult recipients of layperson bystander cardiopulmonary resuscitation by location of cardiac arrest in Pan-Asian communities: A registry-based study
Source: eClinicalMedicine. 2022 Feb 12;44:101293. doi: 10.1016/j.eclinm.2022.101293 (PMC8850341; doi:10.1016/j.eclinm.2022.101293)
Supplement: Supplementary file 1 [file mmc1.docx]

**Supplementary Table 1**. Comparison of cases in the final analysis and cases excluded from final analysis due to missing information. To facilitate comparison, percentage is reported based on the total number of cases with complete information on the corresponding variable.

|  | **Included in final analysis**  **(n=56,192)** | **Excluded from final analysis**  **due to missing information**  **(n=141,160)** | **p-value*** |
| --- | --- | --- | --- |
| **Had BCPR, No. (%)** | 3866 (35.4) | 52,952 (37.5) | <0.001 |
| **Gender, No. (%)** |  |  | <0.001 |
| Female | 19,864 (35.4) | 57,705 (40.9) |  |
| Male | 36,328 (64.6) | 83,443 (59.1) |  |
| Missing | -- | 12 |  |
| **Age, median (Q1-Q3)** | 70 (57, 81) | 75 (60, 84)^#^ | <0.001 |
| **Age, No. (%)** |  |  | <0.001 |
| 18–29 | 1255 (2.2) | 7365 (5.2) |  |
| 30–39 | 2389 (4.3) | 5505 (3.9) |  |
| 40–49 | 4624 (8.2) | 8501 (6.0) |  |
| 50–59 | 8213 (14.6) | 12,093 (8.6) |  |
| 60–69 | 10,450 (18.6) | 20,803 (14.7) |  |
| 70–79 | 13,097 (23.3) | 31,608 (22.4) |  |
| 80+ | 16,164 (28.8) | 55,231 (39.1) |  |
| Missing | -- | 54 |  |
| **Arrest location, No. (%)** |  |  | <0.001 |
| Home residence | 45,278 (80.6) | 14965 (63.8) |  |
| Public/Commercial building | 3984 (7.1) | 1157 (4.9) |  |
| Street/highway | 2532 (4.5) | 4247 (18.1) |  |
| Industrial area | 813 (1.4) | 673 (2.9) |  |
| Transport centre | 256 (0.5) | 115 (0.5) |  |
| Place of recreation | 687 (1.2) | 308 (1.3) |  |
| Other | 2642 (4.7) | 1998 (8.5) |  |
| Missing | -- | 117,697 |  |
| **Time of day, No. (%)** |  |  | <0.001 |
| 11:00 pm – 5:59 am | 10,268 (18.3) | 25026 (18.1) |  |
| 6:00 am – 6:59 pm | 35,881 (63.9) | 89513 (64.7) |  |
| 7:00 pm – 10:59 pm | 10,043 (17.9) | 23740 (17.2) |  |
| Missing | -- | 2881 |  |
| **Witness, No. (%)** |  |  | 0.186 |
| Yes | 24,714 (44.0) | 59,453 (43.7) |  |
| No | 31,478 (56.0) | 76,747 (56.3) |  |
| Missing | -- | 4960 |  |
| **Response time (minutes), median (Q1-Q3)** | 7.0 (5.0, 9.0) | 6.0 (5.0, 8.0) ^^^ | <0.001 |
| **Initial rhythm, No. (%)** |  |  | <0.001 |
| Shockable | 6839 (12.2) | 9501 (6.7) |  |
| Unshockable | 43835 (78) | 116,315 (82.4) |  |
| Cannot determine | 5518 (9.8) | 15,344 (10.9) |  |
| **ROSC at ED, No. (%)** | 15,346 (27.3) | 5052 (3.6) | <0.001 |
| **Survival at discharge or**  **in hospital at day 30, No. (%)** |  |  | <0.001 |
| Yes | 3682 (6.6) | 8066 (5.8) |  |
| No | 52,510 (93.4) | 130,634 (94.2) |  |
| Missing | -- | 2406 |  |
| **Site, No. (%)** |  |  | -- |
| Japan | 13,636 (24.3) | 118,743 (84.1) |  |
| Korea | 12,711 (22.6) | 7505 ( 5.3) |  |
| Malaysia | 1424 (2.5) | 1838 ( 1.3) |  |
| Singapore | 9606 (17.1) | 1279 ( 0.9) |  |
| Taiwan | 14,725 (26.2) | 6765 ( 4.8) |  |
| Thailand | 1046 (1.9) | 663 ( 0.5) |  |
| UAE | 342 (0.6) | 879 ( 0.6) |  |
| China | 696 (1.2) | 717 ( 0.5) |  |
| Philippines | -- | 820 ( 0.6) |  |
| Vietnam | -- | 492 ( 0.3) |  |
| Pakistan | -- | 667 ( 0.5) |  |
| India | 2006 (3.6) | 574 ( 0.4) |  |
| Lebanon | -- | 218 ( 0.2) |  |

*: p-values for comparison between data included and excluded, from the Chi-square test for categorical variables (except for site because different sets of categories were present in the two datasets) and Mann-Whitney test for continuous variables.

#: Excluding 12 cases with missing age.

^: Excluding 3173 cases with missing response time.
